# Etiological distribution and mortality factors in pericardial effusion: Insights from a large cohort study

**DOI:** 10.21542/gcsp.2025.54

**Published:** 2025-10-31

**Authors:** Pooja Vyas Kothari, Kunal Parwani, Kewal Kanabar, Iva Patel, Payal Tripathi, Paritosh Nilesh Kotak, Khushboo Chouhan Parmar

**Affiliations:** U N Mehta Institute of Cardiology and Research Centre, Asarwa, Ahmedabad, Gujarat, India

## Abstract

Background: Pericardial effusion represents a potentially life-threatening clinical entity arising from diverse pathophysiological processes, with etiological prevalence demonstrating substantial geographic and ethnic variation. Despite this recognized heterogeneity, epidemiological data characterizing the distribution of pericardial effusion etiologies in Indian populations remain limited.

Methods: This observational cohort study enrolled 695 consecutive patients presenting with moderate-to-large pericardial effusion at a tertiary cardiac care center. Comprehensive demographic and etiological data were systematically collected, with prospective follow-up extending over a 24-month period.

Results: The 695 patients exhibited a male predominance (55.68%). Etiological analysis revealed tuberculosis as the most frequent cause (32.52%), followed by malignancy (30.6%), idiopathic effusion (19.14%), hypothyroidism (7.77%), chronic kidney disease (5.75%), connective tissue disorders (3.16%), and pyogenic infections (1.0%). Among malignancy-associated effusions, pulmonary carcinoma accounted for 47.41% of cases. In patients presenting with cardiac tamponade, malignancy was identified in 47.12% and tuberculosis in 29.15%. Over the 24-month follow-up period, malignancy was responsible for 78.76% of observed mortality. Kaplan–Meier survival analysis demonstrated that patients with malignancy-associated effusion exhibited the poorest prognosis, followed sequentially by those with chronic kidney disease and tuberculosis.

Conclusion: Tuberculous pericarditis emerged as the predominant etiology of pericardial effusion in this cohort. However, malignancy, particularly pulmonary carcinoma, constituted the principal cause of mortality, followed by chronic kidney disease and tuberculosis. Kaplan–Meier survival analysis underscored the adverse prognostic impact of malignancy-associated pericardial effusion, highlighting the critical importance of prompt diagnostic evaluation and therapeutic intervention.

## Introduction

Pericardial effusion encompasses a broad clinical spectrum, ranging from asymptomatic presentations to life-threatening cardiac tamponade, and is frequently identified as an incidental finding on imaging studies. Despite its prevalence across diverse pathological conditions, current understanding of the incidence, prevalence, and pathophysiological mechanisms governing pericardial fluid accumulation and resorption remains incomplete. The etiology of pericardial effusion typically reflects either systemic disease processes or primary cardiac pathology, with variable rates of fluid accumulation within the pericardial space depending on the underlying condition.

Established etiologies include infectious processes (notably viral, bacterial, and tuberculous pericarditis), malignancy, connective tissue disorders, and pericardial injury syndromes encompassing post-myocardial infarction, post-pericardiotomy, and post-traumatic effusions. Additional recognized causes comprise metabolic derangements such as uremia, hypothyroidism, myopericarditis, aortic dissection, and drug-induced pericarditis^1–3^. Notwithstanding advances in diagnostic evaluation, a substantial proportion of pericardial effusions remain classified as idiopathic following comprehensive investigation^3–5^.

Malignancy-associated pericardial effusion represents the predominant indication for pericardiocentesis in tertiary care settings and frequently constitutes the initial clinical manifestation of underlying neoplastic disease. Malignant effusions are associated with markedly diminished survival compared to effusions of non-malignant etiology^5,6^. Progressive accumulation of pericardial fluid may result in hemodynamic compromise through impaired ventricular filling, culminating in cardiac tamponade, a medical emergency requiring immediate recognition and intervention^7^.

Epidemiological data characterizing the distribution of pericardial effusion etiologies, particularly the relative contributions of primary versus metastatic malignancies, remain sparse and demonstrate considerable geographic and ethnic variability^8,9^.

The present study sought to comprehensively evaluate the etiological spectrum, clinical characteristics, and long-term prognostic outcomes of patients undergoing pericardiocentesis at a tertiary referral center. Through systematic analysis of patient data, we aimed to delineate the underlying causes of pericardial effusion, characterize the clinical presentation and associated features, and identify determinants of patient outcomes.

## Methods

### Study design

This retro-prospective observational study enrolled 695 consecutive patients with moderate-to-large pericardial effusion, with or without cardiac tamponade, who underwent pericardiocentesis at a tertiary cardiac care center. Retrospective data spanning April 2017 to April 2021 were extracted from electronic health records and validated through cross-referencing with discharge summaries and procedural documentation. Prospective data collection was conducted from April 2021 to April 2022 using a standardized protocol and structured case report forms. The study protocol received approval from the Institutional Ethics Committee (UNMICRC/CARDIO/2021/12). Written informed consent was obtained from all prospectively enrolled participants, while the requirement for consent was waived for retrospectively collected data in accordance with institutional guidelines.

### Study population

The study enrolled patients who underwent echocardiographic evaluation and fulfilled diagnostic criteria for pericardial effusion. Inclusion criteria encompassed patients with moderate-to-large pericardial effusion with or without cardiac tamponade, as well as those with mild or loculated effusion associated with tamponade physiology.

Exclusion criteria comprised: (1) age less than 18 years; (2) incomplete medical documentation, including inadequate pericardial fluid analysis, incomplete laboratory evaluation precluding definitive etiological diagnosis, or absent mortality follow-up data; and (3) iatrogenic or post-traumatic pericardial effusion. The exclusion of traumatic effusions was implemented to enhance cohort homogeneity, as these effusions result from procedural interventions or mechanical injury rather than infectious or neoplastic processes, thereby representing a distinct pathophysiological entity.

Pericardial effusion was diagnosed by transthoracic echocardiography, characterized by an echo-free space in the pericardial cavity. Effusion severity was classified according to maximal diastolic echo-free space: small (<10 mm), moderate (10–20 mm), and large (>20 mm). Effusions measuring less than 10 mm without hemodynamic compromise were excluded from analysis.

Cardiac tamponade was defined by the presence of pericardial effusion with echocardiographic evidence of chamber collapse (right ventricular early diastolic collapse or right atrial systolic collapse) and respiratory variation in transvalvular flow velocities (>25% variation in mitral inflow or > 40% variation in tricuspid inflow), accompanied by clinical signs including tachycardia, hypotension, or pulsus paradoxus.

Comprehensive etiological investigation included complete blood count with erythrocyte sedimentation rate, renal function tests (blood urea and serum creatinine), tuberculin skin testing, chest radiography, thyroid function profile, antinuclear antibody, rheumatoid factor, and computed tomography of the chest.

Pericardial fluid obtained during pericardiocentesis underwent systematic analysis including total and differential cell counts, protein and lactate dehydrogenase levels, cytological examination for malignant cells, adenosine deaminase activity, polymerase chain reaction for *Mycobacterium tuberculosis*, Gram staining, acid-fast bacilli staining, and bacterial and mycobacterial cultures.

Definitive etiological diagnosis was established through integration of clinical presentation, physical examination findings, and targeted laboratory investigations specific to tuberculosis, uremia, malignancy, and connective tissue disorders. Therapeutic pericardiocentesis was performed under continuous echocardiographic guidance using percutaneous catheter drainage via either subxiphoid or apical approach, with placement of a pigtail catheter in the pericardial space.

### Definition of diagnosis

Tuberculous pericardial effusion was diagnosed based on one or more of the following criteria: positive acid-fast bacilli staining or culture from pericardial fluid, positive polymerase chain reaction for *Mycobacterium tuberculosis* in pericardial fluid, elevated adenosine deaminase level (>40.1 IU/L) in lymphocyte-predominant effusion, and/or radiographic evidence of pulmonary tuberculosis on computed tomography.

Malignancy-associated pericardial effusion was established by histopathological or cytological evidence of malignancy on tissue biopsy or imaging studies, irrespective of prior or concurrent oncological treatment (radiotherapy or chemotherapy), and/or identification of malignant cells on pericardial fluid cytological examination.

Chronic kidney disease was defined as documented renal dysfunction with abnormal renal function parameters, with or without renal replacement therapy.

Connective tissue disease was diagnosed in patients with established autoimmune disorders, whether treatment-naïve or receiving immunosuppressive therapy, demonstrating compatible clinical manifestations and positive autoimmune serological markers.

Idiopathic or presumed viral pericarditis was diagnosed by exclusion, based on characteristic clinical presentation in the absence of identifiable etiology following comprehensive diagnostic evaluation.

### Follow up

Long-term survival outcomes were ascertained through structured telephonic follow-up conducted at 24 months post-index admission. During these contacts, standardized data were collected regarding vital status, clinical course, and relevant complications. This methodology facilitated systematic assessment of patient outcomes beyond the acute hospitalization period.

### Statistical analysis

Statistical analyses were performed using SPSS version 26.0 software (IBM Corporation, Chicago, IL, USA). Categorical variables were expressed as frequencies and percentages, while continuous variables were presented as mean ± standard deviation. Between-group comparisons of continuous variables were conducted using Student’s *t*-test, and categorical variables were analyzed using the chi-square test. Kaplan–Meier survival analysis was performed to evaluate long-term survival stratified by etiological category. The diagnostic performance of adenosine deaminase levels for tuberculous pericardial effusion was assessed through receiver operating characteristic curve analysis, with calculation of sensitivity, specificity, positive predictive value, and negative predictive value. Statistical significance was defined as a two-tailed *P* value < 0.05.

## Results

Table 1 summarizes the demographic and clinical characteristics of the study cohort. The mean age was 48.03 ± 16.68 years, with male predominance (55.68% vs. 44.32%). The most common presenting symptoms included dyspnea (19%), chest pain (10.8%), fever (9.7%), and cough (7.3%). Electrocardiographic abnormalities were observed in a substantial proportion of patients, with tachycardia noted in 53.5%, electrical alternans in 21.7%, and low voltage in 40.14%.

**Table 1:** Baseline clinical characteristics.

**Variables**	**N (%)/** **Mean ± SD (*N* = 695)**
Age	48.03 ± 16.68
Male	387 (55.68%)
Female	308 (44.32%)
**Clinical presentation**	
Shortness of breath	316 (45.47%)
Chest pain	76 (10.8%)
Fever	68 (9.7%)
Cough	51 (7.3%)
**ECG**	
Tachycardia	372 (53.5%)
Electric alterans	151 (21.7%)
Low voltage	279 (40.14%)
**Echocardiographic parameters**	
Large PE	546 (78.56%)
Moderate PE	149 (21.44%)
Cardiac Tamponade	295 (42.43%)
**Type of effusion**	
Circumferential	605 (87.05%)
Loculated	90 (12.9%)

Echocardiographic assessment revealed large pericardial effusion in 78.56% and moderate effusion in 21.44% of patients. Cardiac tamponade was present in 42.43% of the cohort. Morphologically, the majority of effusions were circumferential (87.05%), with loculated effusions comprising 12.9% of cases.

Table 2 presents the etiological spectrum of pericardial effusion in the study cohort. Tuberculosis was the most prevalent etiology (32.52%), followed by malignancy (30.6%) and idiopathic effusion (19.14%). Less frequent causes included hypothyroidism (7.77%), chronic kidney disease (5.75%), connective tissue disorders (3.16%), and pyogenic infections (1%).

**Table 2:** Etiology of study patients.

**Variables**	**Number (%) (*N* = 695)**
Tuberculosis	226 (32.52%)
Malignancy	213 (30.6%)
Idiopathic	133 (19.14%)
Hypothyroidism	54 (7.77%)
CKD	40 (5.75%)
CTD	22 (3.16%)
Pyogenic	7 (1%)

Among the 213 patients with malignancy-associated effusion (30.6% of total cohort), the primary tumor distribution was as follows: lung carcinoma (47.41%), breast carcinoma (24.88%), lymphoma (21.6%), and leukemia (6.1%).

In the subgroup of 295 patients presenting with cardiac tamponade (42.43% of total cohort), the etiological distribution differed notably: malignancy accounted for 47.12%, tuberculosis for 29.15%, idiopathic causes for 10.17%, hypothyroidism for 6.77%, chronic kidney disease for 5.76%, and pyogenic infections for 1.02%.

**Figure 1.**
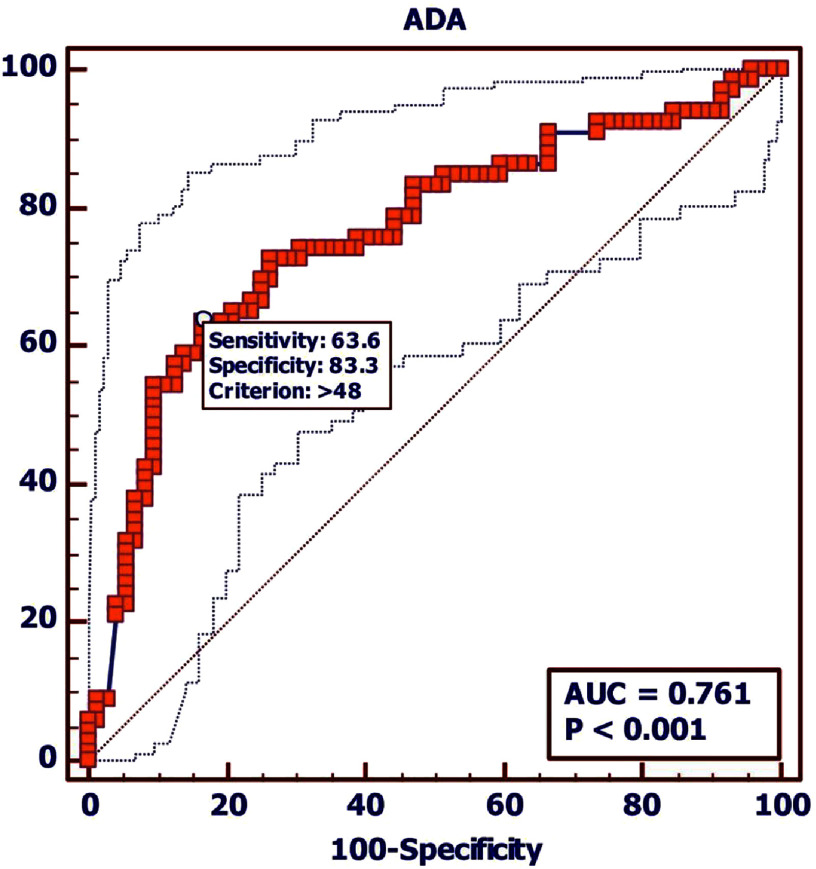
Receiver operating characteristic curve analysis.

Elevated adenosine deaminase levels (>40.1 U/L) demonstrated significant association with tuberculous pericardial effusion (*p* < 0.0001). Receiver operating characteristic curve analysis yielded an area under the curve of 0.811 (95% confidence interval: 0.75–0.86, *p* < 0.0001), with corresponding sensitivity of 73.5% and specificity of 82.1% (Figure 1).

Among patients with confirmed tuberculous pericardial effusion (*N* = 226), 73.45% demonstrated adenosine deaminase levels exceeding 40.1 U/L, compared with 18.34% in the non-tuberculous group (*N* = 469), representing a statistically significant difference (*p* < 0.0001). Conversely, adenosine deaminase levels below the threshold of 40.1 U/L were observed in 26.55% of tuberculous cases and 81.66% of non-tuberculous cases.

Table 3 summarizes mortality data during the 24-month follow-up period. Overall, 113 patients died, yielding a cumulative mortality rate of 16.26%. The etiological distribution among deceased patients was: malignancy (78.76%), chronic kidney disease (12.39%), and tuberculosis (8.84%).

**Table 3:** Etiology in patients with mortality.

**Mortality (*n* = 113, 16.26%)**	**Number (%)**
Malignancy	89 (78.76%)
Chronic kidney disease (CKD)	14 (12.39%)
Tuberculosis (TB)	10 (8.84%)

Among the 89 malignancy-related deaths (12.8% of the total cohort), mortality was stratified by primary tumor type as follows: lung carcinoma (7.19%), breast carcinoma (2.88%), lymphoma (1.87%), and leukemia (1%).

**Table 4:** Pairwise Log Rank (Mantel-Cox) comparison with of etiology associated mortality. TB-Tuberculosis; CKD-Chronic kidney disease; P value ≤0.05 shows statistical significance.

**Etiology**	**Chi-Square**	**95% CI lower-upper**	**P-value**
TB vs Malignancy	14.523	1.25 to 5.00	<0.0001
Malignancy vs. CKD	0.207	0.47 to 3.04	0.649
CKD vs. Malignancy	3.131	0.73 to 5.46	0.077

**Figure 2.**
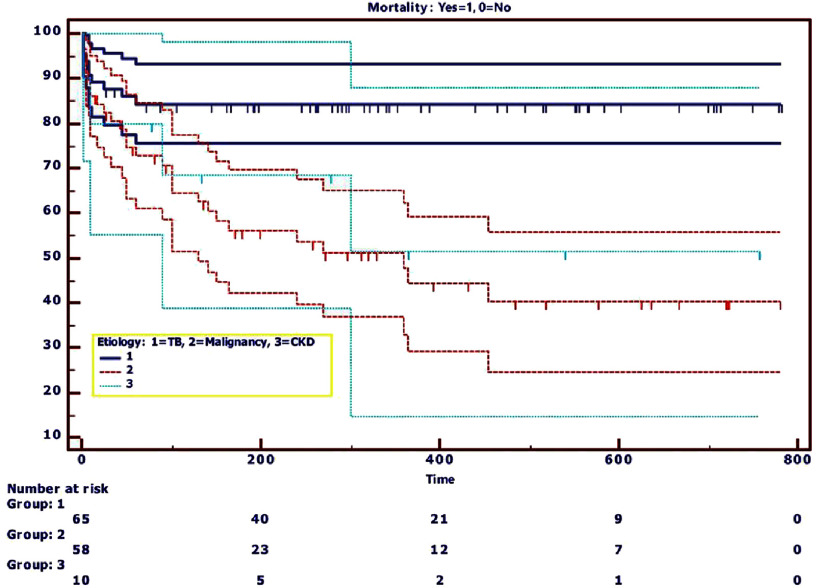
Kaplan–Meier survival analysis stratified by etiology.

Kaplan–Meier survival analysis stratified by etiology demonstrated significant prognostic heterogeneity (Figure 2). Malignancy-associated pericardial effusion was associated with the poorest survival outcomes, followed by chronic kidney disease, while tuberculosis conferred relatively favorable long-term survival. The survival difference between malignancy-associated and tuberculous pericardial effusion reached statistical significance (*p* < 0.0001, Table 4).

Pairwise log-rank (Mantel-Cox) analysis (Table 4) revealed significant mortality differences between tuberculous and malignancy-associated pericardial effusion (*χ*^2^ = 14.523, *p* < 0.0001; 95% confidence interval: 1.25–5.00). No statistically significant survival differences were identified between malignancy and chronic kidney disease (*χ*^2^ = 0.207, *p* = 0.649; 95% confidence interval: 0.47–3.04) or between chronic kidney disease and tuberculosis (*χ*^2^ = 3.131, *p* = 0.077; 95% confidence interval: 0.73–5.46).

**Table 5:** Cox proportional hazards analysis. Pairwise comparisons of mortality risk by etiology. Each cell represents the hazard ratio (HR) with 95% confidence interval (CI) for the row etiology compared to the column etiology as reference. HR >1 indicates increased mortality risk; HR <1 indicates decreased mortality risk relative to the reference category.

**Etiology**	**Tuberculosis**	**Malignancy**	**Chronic Kidney Disease**
	**HR (95% CI)**	**HR (95% CI)**	**HR (95% CI)**
**Tuberculosis**	**–**	**3.67 (1.95–6.91)**	**2.92 (0.88–9.71)**
**Malignancy**	**0.28 (0.14–0.51)**	**–**	**0.80 (0.23–2.71)**
**Chronic Kidney Disease**	**0.34 (0.10–1.14)**	**1.26 (0.37–4.27)**	**–**

**Table 6:** Univariate and multivariable Cox regression analysis of mortality predictors. Variables not included in the multivariable model are indicated by dashes (–). Large pericardial effusion was included in the multivariable model but did not retain statistical significance.

**Variable**	**Univariate analysis**			**Multivariable analysis**		
	**HR**	**95% CI**	***P*** **-value**	**HR**	**95% CI**	***P*** **-value**
Age (per year)	1.024	1.005–1.04	0.01	1.02	1.00–1.04	0.056
Male gender	0.72	0.38–1.36	0.31	–	–	–
Large pericardial effusion	2.79	0.86–9.07	0.09	0.52	0.14–1.97	0.33
Pericardiocentesis	0.90	0.44–1.83	0.76	–	–	–
Cardiac tamponade	20.59	6.28–67.53	<0.0001	26.22	6.85–100.35	<0.0001

Cox proportional hazards analysis (Table 5) confirmed that etiology was significantly associated with mortality (overall omnibus Wald *χ*^2^ = 14.813, df = 2, p 0.001). Compared with TB, malignancy was associated with a 72% lower hazard of death (HR = 0.28, 95% CI: 0.14–0.51), while CKD showed a non-significant reduction (HR = 0.34, 95% CI: 0.10–1.14). When malignancy was the reference, TB was associated with a 3.67-fold increased hazard (95% CI: 1.95–6.91), whereas CKD had no significant difference in hazard (HR = 1.26, 95% CI: 0.37–4.27).

Univariate Cox regression analysis (Table 6) identified increasing age (HR = 1.024 per year, 95% CI: 1.005–1.04, *p* = 0.01) and presence of cardiac tamponade (HR = 20.59, 95% CI: 6.28–67.53, *p* < 0.0001) as significant predictors of mortality. In the multivariable model adjusting for potential confounders, cardiac tamponade remained a robust independent predictor of mortality (HR = 26.22, 95% CI: 6.85–100.35, *p* < 0.0001), while the association with age demonstrated borderline statistical significance (HR = 1.02, 95% CI: 1.00–1.04, *p* = 0.056). Gender, effusion size (large vs. moderate), and pericardiocentesis were not independently associated with mortality outcomes.

## Discussion

Long-term survival in patients with pericardial effusion is significantly influenced by underlying etiology. The etiological spectrum demonstrates substantial geographic variability, reflecting regional differences in disease prevalence. Clinical presentations range from asymptomatic effusions to life-threatening cardiac tamponade. Accurate etiological diagnosis, particularly identification of malignancy, metastatic disease, or bacterial infection, represents a critical determinant of prognosis and long-term outcomes. However, contemporary epidemiological data characterizing the distribution of pericardial effusion etiologies, particularly in the Indian subcontinent, remain limited. Furthermore, the considerable effect of these underlying causes on patient outcomes is underestimated.

The present study represents one of the largest Indian cohorts to date, comprising 695 consecutive patients with comprehensive etiological characterization and outcome data. Prior investigations have documented distinct etiological patterns of pericardial effusion between developed and developing nations.

The present analysis revealed tuberculosis as the predominant etiology of pericardial effusion (32.52%), followed by malignancy (30.6%), idiopathic causes (19.14%), hypothyroidism (7.77%), chronic kidney disease (5.75%), connective tissue disorders (3.16%), and pyogenic infections (1%). Cardiac tamponade was identified in 42.43% of patients, with diverse underlying etiologies.

Among patients presenting with tamponade, malignancy predominated (47.12%), followed by tuberculosis (29.15%), idiopathic causes (10.17%), hypothyroidism (6.77%), uremia (5.76%), and pyogenic infections (1.02%). Previous reports have documented tamponade rates of 77.8% in unselected pericardial effusion cohorts^10^ and 54.13% among oncology patients requiring pericardiocentesis^11^.

Analysis of the 2016–2019 Nationwide Readmissions Database identified cardiac tamponade in 62% of patients undergoing pericardiocentesis^12^. This substantial variation in tamponade incidence across populations likely reflects multiple factors, including ethnic diversity, geographic location, and differing etiological distributions. Regional disparities in disease prevalence, healthcare access, and diagnostic practices may further contribute to this heterogeneity. The underlying etiology - particularly malignancy, tuberculosis, or metabolic disorders - appears to significantly influence tamponade development, thereby contributing to the observed epidemiological variability.

Recent Indian studies have reported tuberculous etiology in 17%^13^ and 26.8%^14^ of pericardial effusion cases. Tuberculosis remains the predominant cause of pericardial effusion in low- and middle-income countries, accounting for 40–70% of cases^15^. In the present cohort, tuberculous pericarditis was identified in 32.52% of patients. The global burden of tuberculous pericardial disease is concentrated in southern Asia, sub-Saharan Africa, and the western Pacific, which collectively represent approximately 87% of cases worldwide^16,17^. Our findings, in conjunction with other Indian data^13^, suggest that despite India’s classification as a lower-middle-income country, the prevalence of tuberculous pericardial effusion may be lower than the global average for this economic stratum.

This relatively lower incidence of tuberculous pericardial effusion may reflect improved public health infrastructure and enhanced tuberculosis control programs, particularly in rural areas, alongside evolving disease patterns in urban tertiary care settings. Additionally, referral bias inherent to our specialized cardiac center, which predominantly manages complex oncological cases, may have augmented malignancy representation while potentially underestimating the true burden of tuberculous pericarditis in the broader population.

In the present cohort, adenosine deaminase levels exceeding 40.1 U/L demonstrated significant association with tuberculous pericardial effusion, consistent with prior Indian data from Manipal^13^. The pathogenesis of tuberculous pericarditis encompasses multiple mechanisms, including direct pericardial infection, immune-mediated pericardial inflammation, and lymphohematogenous dissemination of mycobacterial disease.

Previous Indian studies have reported substantially lower malignancy rates: 13.63% in Bihar^18^ and 14% in Kashmir^19^. In contrast, the present study identified malignancy in 30.6% of patients with pericardial effusion. This disparity likely reflects referral bias, as our tertiary cardiac center receives a disproportionate volume of complex oncological cases requiring specialized pericardial intervention.

The distribution of primary malignancies in our cohort was: lung carcinoma (47.41%), breast carcinoma (24.88%), lymphoma (21.6%), and leukemia (6.10%). This contrasts with the findings of Gupta et al.^20^, who reported Hodgkin’s disease, lymphoma, and leukemia as the predominant malignancy-associated etiologies.

In the present cohort, malignancy accounted for the majority of deaths (78.76%), followed by chronic kidney disease (12.39%) and tuberculosis (8.84%). Kaplan–Meier survival analysis demonstrated significantly inferior survival in malignancy-associated pericardial effusion compared to tuberculous and chronic kidney disease etiologies. These findings are consistent with prior reports documenting poor prognosis in malignant pericardial disease^6,21^. Pericardial metastasis represents an adverse prognostic marker, particularly in patients without prior oncological diagnosis^6^.

The idiopathic category comprised 19.14% of cases in the present cohort, raising considerations regarding diagnostic completeness in real-world clinical practice. Despite implementation of structured diagnostic protocols - including comprehensive pericardial fluid analysis, autoimmune serological evaluation, and advanced imaging - resource limitations may have contributed to underdiagnosis of specific etiologies. A single-center Japanese study by Hori et al. reported idiopathic cases as high as 32%^4^, while our findings align with the broader literature documenting idiopathic pericardial effusion rates of 10–30%, even in well-resourced healthcare settings.

### Limitations of the study

This study has several limitations. As a single-center investigation, external validity and generalizability are inherently constrained. The tertiary referral nature of our institution may have enriched the cohort with severe and complex cases, further limiting broader applicability. The findings are specific to pericardial effusion of medical etiology and therefore not generalizable to traumatic effusions. Future investigations would benefit from inclusion of control groups without pericardial effusion to better elucidate the relationship between comorbidities and disease development. The retrospective data collection methodology for the initial study period may introduce selection and information bias. Finally, the sample size and regional focus may limit extrapolation to other geographic and demographic populations.

### Practical implications and recommendations

• Pericardial effusion with adenosine deaminase levels exceeding 40.1 IU/L in the context of lymphocyte-predominant exudate should prompt strong clinical suspicion for tuberculous etiology.
• Given the substantial mortality risk associated with malignant pericardial effusion, aggressive diagnostic evaluation incorporating advanced imaging and comprehensive cytological analysis is warranted.
• Cardiac tamponade represents a medical emergency; accordingly, all healthcare facilities should maintain echocardiographic capabilities and ensure primary care physicians possess competency in pericardial effusion detection.
• Patients presenting with moderate-to-large pericardial effusion accompanied by tamponade physiology require expedited triage for emergent pericardiocentesis.
• Systematic long-term follow-up extending to 24 months provides essential prognostic information and should be incorporated into standard care protocols across all healthcare settings.

## Conclusion

Tuberculosis represented the predominant etiology of pericardial effusion in this cohort, with adenosine deaminase levels exceeding 40.1 U/L demonstrating strong diagnostic association with tuberculous pericarditis. Malignancy, particularly lung carcinoma, emerged as the principal cause of mortality, followed by chronic kidney disease and tuberculosis. Kaplan–Meier survival analysis confirmed significantly reduced survival in patients with malignancy-associated pericardial effusion. These findings emphasize the critical importance of early etiological diagnosis and targeted therapeutic intervention, particularly for malignant and tuberculous disease, to optimize patient outcomes. The results underscore the necessity of comprehensive diagnostic evaluation in the management of pericardial effusion.

While constrained by single-center design and partially retrospective methodology, this study provides important insights into the clinical spectrum, outcomes, and prognostic determinants of pericardial effusion in a lower-middle-income country setting. Future prospective, multicenter investigations are warranted to validate these observations, refine diagnostic algorithms, and establish cost-effective, etiology-directed management strategies.
